# Spatial occurrence-intensity modeling of dengue incidence in southernmost provinces of Thailand

**DOI:** 10.1371/journal.pntd.0013347

**Published:** 2025-07-23

**Authors:** Apiradee Lim, Lumpoo Ammatawiyanon, Haris Khurram, Phattrawan Tongkumchum, Don McNeil

**Affiliations:** 1 Department of Mathematics and Computer Science, Faculty of Science and Technology, Prince of Songkla University, Pattani Campus, Pattani, Thailand; 2 Department of Sciences and Humanities, National University of Computer and Emerging Science, Chiniot-Faisalabad Campus, Chiniot, Pakistan; Faculty of Science, Ain Shams University (ASU), EGYPT

## Abstract

**Background:**

Dengue is currently spreading and is considered a hyperendemic in Thailand. Outbreaks happen almost every year in various provinces of Thailand, especially the four southernmost provinces consistently reporting for more than a decade. This study aimed to explore the spatial distribution and high-risk areas using an occurrence-intensity model at a sub-district level in the four southernmost provinces of Thailand.

**Methods:**

The record of the cases admitted to the hospital and diagnosed as dengue in the 377 sub-districts of four southernmost provinces, Songkhla, Pattani, Yala, and Narathiwat from 2008 to 2020, gender-age wise, were taken from the Office of Disease Prevention and Control, Ministry of Public Health, Thailand. We suggested a methodology based on the occurrence-intensity model to analyze the dengue cases in two steps. At first, the occurrence is determined by using the logistic regression model and considering the variable of interest as a case or not. While at second, the intensity is determined by fitting a log-linear regression model for disease intensity after excluding zeros.

**Results:**

The results from 78,416 observations revealed that a total number of 68,526 dengue cases were registered from 2008-2020 in all four southernmost provinces. The overall average occurrence rate was 28.3% while the average intensity was 419 per 100,000 population. The occurrence-intensity model gives a much better fit to the data and highlights that the gender-age patterns of occurrence and intensity are different. Occurrence is higher among young adult ages and then declines with age for each gender, whereas intensity is higher in children, young adults, and the elderly for each gender. The sub-districts are in the suburbs of the Songkhla province and sporadically, areas on the border of the Narathiwat province had high occurrence and intensity.

**Conclusions:**

The spatial occurrence and intensity of dengue in sub-districts can provide valuable guidance to identify high-risk areas and monitor the intensity of dengue cases in these areas. This will be useful for healthcare departments in developing effective public health strategies for dengue control.

## Author summary

Dengue has dramatically spread globally, which occurs almost every year, especially in the topical areas. In Thailand, the outbreak of dengue occurs almost every year. The spatial distribution of the outbreak of the areas on a small scale has not been investigated in the southernmost provinces. Using the concept of the combination of occurrence and intensity of the disease is an effective method to capture the hot spot areas by understanding the pattern of spreads and also the areas that need to be focused on by health experts to eliminate the spread of the disease. The results from this study revealed that the occurrence decreased with age, but the intensity was high in adolescents and the elderly. During the period of 2008–2021 three episodes occurred. Most of the hot spot areas were the urban districts in Songkhla and Narathiwat with some districts in the border. The study suggests that identifying high-risk areas and monitoring dengue cases can help healthcare departments develop effective public health strategies for dengue control.

## Introduction

The global incidence of dengue has grown dramatically in recent decades. About 3.9 billion of the world’s population annually is to be at risk [1]. Each year, about 500,000 people require hospitalization and around 20,000 people die due to severe dengue fever [2,3]. Dengue virus (DENV) has four antigenically distant serotypes that infect humans with *Aedes aegypti* and *Aedes albopictus* mosquito vectors [4]. The first isolation of DENV was in 1943. After about 60 years from the first isolation, it spread globally to almost every continent [5]. Dengue is considered a major cause of morbidity and mortality in tropical and sub-tropical countries. Cattarino [6] and Paz-Bailey [7] reported that Southeast Asia is one of the highly endemic global regions. This causes a substantial burden on the healthcare system and households.

Dengue is in active circulation in Thailand and is considered a hyperendemic with about 100,000 cases reported annually [8,9]. Also proposed a the spatial distribution and clustering of dengue outbreaks in different regions of Thailand were discussed in the literature [10–12]. Many researchers study the association between climate and other weather-related variables on dengue fever and its surveillance in different regions of Thailand [13–15]. Thailand has a robust national dengue surveillance system, which has provided an enormous amount of data on the burden of dengue in the country. The findings of dengue incidence on a small scale are relatively scarce compared to those available on a larger scale [16]. Understanding the spatial pattern of dengue in sub-districts of the provinces and identifying those areas with high incidence rates are essential for the wise allocation of limited public health resources [17]. However, existing studies often focus on provincial levels in central, northern, and northeast Thailand or discuss the overall country level. Very few studies have been conducted in southern Thailand, especially in the southernmost provinces of Thailand. These four southernmost provinces have reported non-zero cases for over a decade and have tropical weather. So, it is important to understand the pattern of dengue outbreaks in these regions. Moreover, to understand the patterns and spatial distribution of dengue incidence is needed a methodology that can manage data with both zero and non-zero cases and identify the patterns that help experts and policymakers to identify the hotspots for earlier prevention.

The present study aims to explore the spatial distribution and high-risk areas of dengue occurrence and intensity at a fine scale of sub-districts of four southernmost provinces of Thailand. We also proposed a methodology based on an occurrence-intensity model which is a two-step modeling approach for analyzing the dengue patterns. Our methodology, at the first step, measures the probability of the occurrence of dengue incidences in sub-districts and then evaluates the intensity of the dengue cases in each sub-district. This will help to identify and rank the high-risk areas in order to monitor intensively and make policies for reducing the occurrence and intensity of dengue cases.

## Materials and methods

### Ethics statement

This study has been approved by the Human Research Ethics Committee of Prince of Songkla University, Pattani Campus, under approval number psu.pn 1–007/63. The need for informed consent was waived by the Human Research Ethics Committee of Prince of Songkla University due to the study’s retrospective nature and the absence of patient identifiers in the presented data.

### Study area

**Fig 1:**
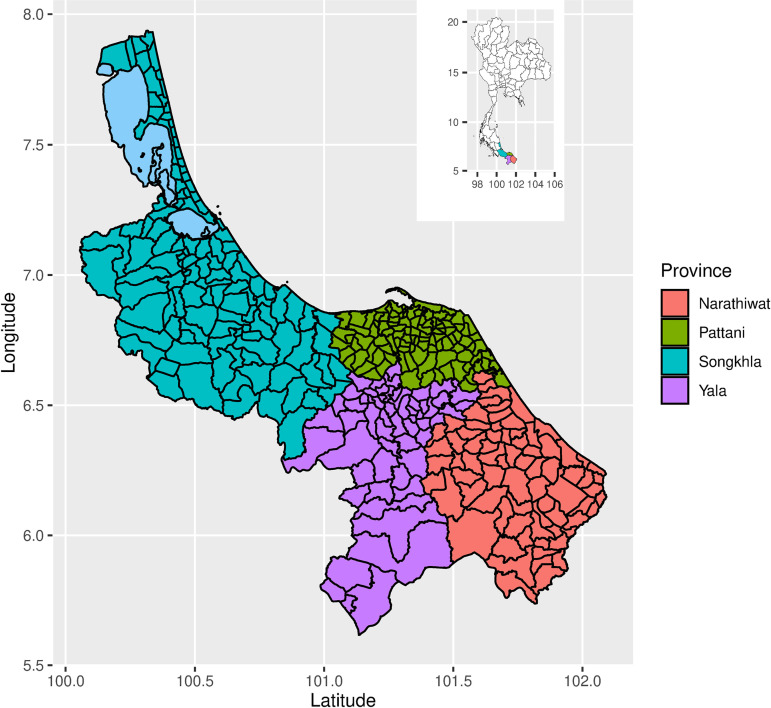
The four southernmost provinces of Thailand.

The provinces of Thailand can be classified into five subnational regions in accordance with their climatic characteristics. The southern Thailand region consists of 14 provinces, including the four southernmost provinces: Songkhla, Pattani, Yala, and Narathiwat. The study areas covered these four southernmost provinces of Thailand. These four provinces had non-zero reports of dengue incidence for the last ten years. In Thailand, the administrative division levels include province, district, sub-district, and village. The four southernmost provinces of Thailand collectively encompass 377 sub-districts, with a total population of 3.5 million in 2021 and 18,330 square kilometers area. These provinces are located around 1,000 kilometers south of the capital city, Bangkok. Fig 1 shows a map depicting the four southernmost provinces with sub-districts in Thailand.

### Data description

Dengue is a disease that requires to be notified by the physicians. According to the National Communicable Disease Control Law, physicians are responsible for diagnosing suspected cases and categorizing them based on the severity of the disease. Dengue is categorized as: Dengue fever (DF), dengue hemorrhagic fever (DHF), and dengue shock syndrome (DSS). However, the specific serotype is not usually documented.

Data of dengue cases in all disease codes from 2008 to 2020 was obtained from the Office of Disease Prevention and Control, Ministry of Public Health. The patients’ ages were already divided into eight groups based on 10-year intervals: 0–9, 10–19, 20–29, 30–39, 40–49, 50–59, 60–69, and 70 and above. Gender and age were combined into a single variable with sixteen levels to observe the effect of gender-age-wise incidence rates. The data were organized in the form of a table where each observation was the number of diseases by subdistrict, gender-age group, and year. Data on dengue case count was utilized to analyze the patterns of gender-age-wise dengue outbreaks in all sub-districts of four southernmost provinces from 2008 to 2020. Thus for 377 sub-districts, 16 categories of gender-age groups, and 13 years, we have a total of 78,416 data records.

### Statistical analysis

We used boxplot and thematic map to explore the distribution of dengue in different sub-divisions at different years and gender-age groups. After that, to evaluate the incidence, we used a two-step approach for modeling which we called the occurrence-intensity model.

Incidences were the number of cases reported from each sub-district. Incidences may have zero or greater than 1 for a specific sub-district and category. To manage zeros in incidences, we used occurrence modeling. Occurrence was the gender-age and year-wise adjusted percentage of at least one dengue case in a sub-district as $Percetange=\frac{odds}{1+odds}\times 100$. While intensity was the number of cases after excluding zero.

So, firstly, we considered disease occurrence as a binary outcome coded 0 when there was no dengue record and 1 when there was at least one dengue record. Then we fitted a logistic regression model to evaluate the probability of the occurrence for gender-age groups, year and sub-districts as predictors. To account for differences in population sizes among different sub-districts and further allow interpretability of the regression coefficients, we include the population size group as an additional predictor. Which was categorized as less than 400, 400–599, 600–799, and 800 or more. Through logistic regression, we estimated the logit of the probability of dengue occurrence as a linear function of the predictors. The logistic regression model with four predictors is formulated as:


$$\ln\left(\frac{p_{ijkl}}{1-p_{ijkl}}\right)=\mu +\alpha_{i}+\beta_{j}+\delta_{k}+\gamma_{l}$$ (1)

where $p_{ijkl}$ is the probability of dengue occurrence in a combination of *μ* a constant, $\alpha_{i}$, $\beta_{j}$, $\delta_{k}$ and $\gamma_{l}$ as effect terms specifying gender-age *i*, year *j*, sub-districts *k,* and population group *l*, respectively. To assess the accuracy of model prediction, the Receiver Operating Characteristic (ROC) curve from logistic regression was drawn. The area under the ROC curve (AUC) evaluates the performance of the model in terms of its accuracy.

Secondly, we model the intensity of dengue incidence which was a conditional incidence after excluding records with no cases. We fit a linear model to the log-transformed intensity rates. This will explore where and how much sickness manifests itself. A log-linear model was used for the intensity rates with gender-age group, year, and sub-district as predictors. Through log-linear regression, we estimated the intensity as a linear function of the factors. The log-linear regression model with three factors is formulated as,


$$\ln\left(\frac{n_{ijk}}{P_{ijk}}\right)=y_{ijk}=\mu +\alpha_{i}+\beta_{j}+\delta_{k}$$ (2)

where $n_{ijk}$ is the corresponding number of reported cases and $P_{ijk}\;$ is the corresponding population. A quantile-quantile (Q-Q) plot of studentized residuals was used to assess the performance of the model.

The models were fitted using sum contrasts as suggested by [18] instead of conventional treatment contrasts where the first category of each predictor was left out from the model as the reference. These methods allow us to compute the estimate and its 95% confidence interval for both dengue occurrences and intensity rates for each factor level adjusted for other factors. A plot of adjusted estimates with 95% confidence intervals for each level of the predictors was created using the results of these models. The thematic map was subsequently developed by categorizing results based on whether their occurrence and intensity rates fall above, across, or below the overall mean. R language was used for all statistical analysis and graphical displays.

## Results

**Fig 2:**
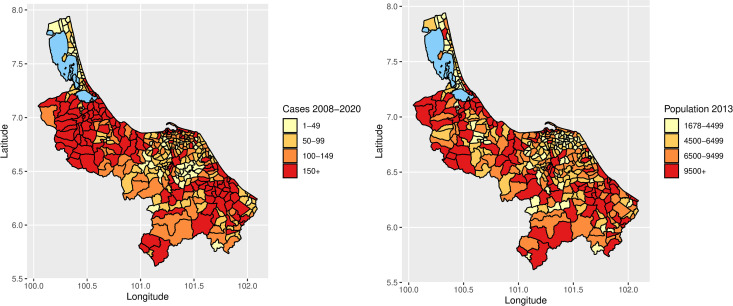
Thematic maps of number of dengue cases in 2008-2020 and the size of the population in 2013 by sub-districts of the four southernmost provinces of Thailand.

A total of 68,526 dengue cases were reported in the four southernmost provinces during the thirteen-year duration; these cases were dispersed among all sub-districts. Fig 2 shows thematical maps for dengue cases and population size for each sub-district. The highest number of cases reported was 4,733 in Hat Yai, a sub-district of Songkhla. The highest and lowest annual population was 148,284 in 2013 in Hat Yai, a sub-district of Songkhla, and 1,678 in Ta Che, a sub-district of Yala, respectively.

**Fig 3:**
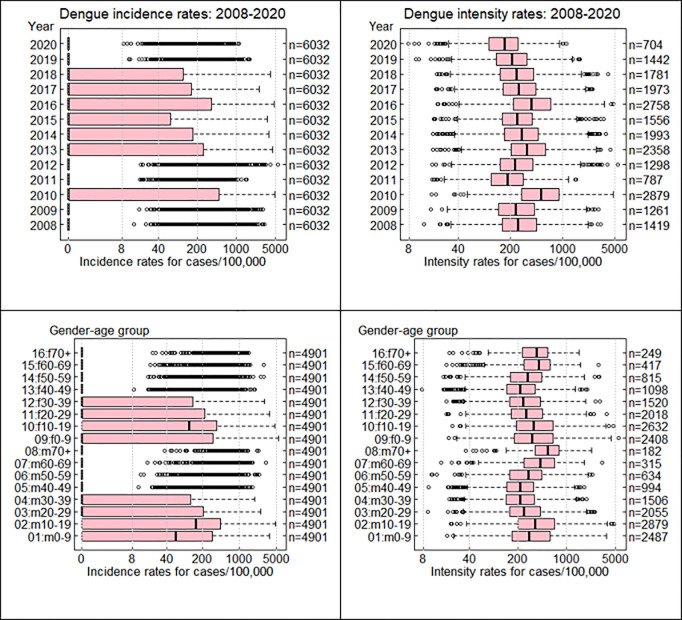
Box plots of incidence rates (on the left panel) and intensity rates (on the right panel) for years and gender-age-wise comparison.

**Fig 4:**
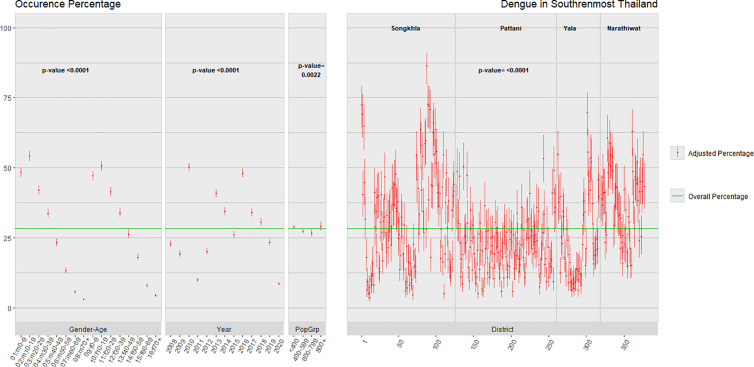
Dengue occurrence in 2008-2020 for levels of each determinant including cell population size group in southernmost provinces of Thailand. The overall Mean is the Overall Mean of the Percentages.

Fig 3 shows boxplots for incidence and intensity rates of dengue by gender-age and year group. The boxplots of the incidence rate predominantly exhibit outliers because the third quartile of dengue incidence for the specified age group is zero. Out of a total of 78,416 data records, 22,209 exhibited a dengue occurrence, resulting in an overall occurrence rate of 28.3%. Throughout the study period, there was a peak occurrence of 47.7% in 2010, followed by 45.7% in 2016 and 39.1% in 2013. The intensity rate has a consistent pattern, with notable peaks in 2010, 2013, and 2016. Each gender-age group exhibited distinct patterns of occurrence and intensity. The occurrence declines with age for both sexes, however, high-intensity rates were noted in young children, adolescents, and adults 60 years of age and beyond. Fig 4 depicts the ROC curve and Q-Q plots of the deviance residuals of the log-linear model. The AUC for the logistic model is 71.94% showing that the model is an adequate fit. Similarly, the Q-Q plot of the log-linear model suggests that the model is a good fit.

The 95% confidence intervals for dengue occurrence by gender-age group, year, and sub-district are shown in Fig 4. The overall mean percentage occurrence was 28.3% as shown by the horizontal red line. Age patterns indicate a notable peak during the teenage years for males, a slightly lower peak for females, and a decline with increasing age for both genders.

There was a noticeable variation among sub-districts, particularly in Songkhla and Narathiwat provinces, where there were areas with high occurrence. Interestingly, there was also a noticeable decrease in occurrence within a population size group.

**Fig 5:**
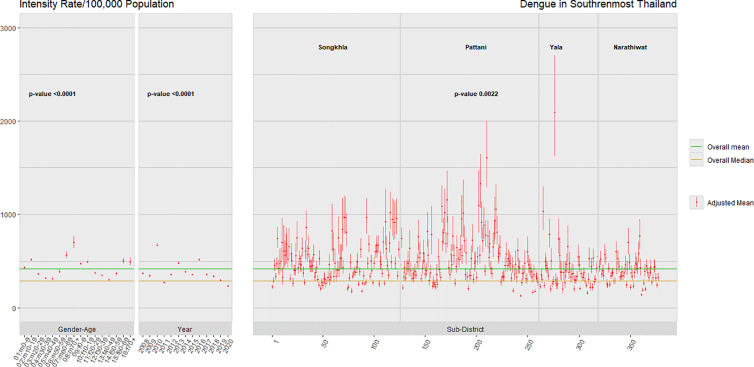
Dengue intensity in 2008-2020 for levels of each determinant factor in southernmost provinces of Thailand.

Fig 5 displays the confidence intervals of dengue intensity corresponding to different demographic risk factors. The overall median is 285 cases per 100,000 population, while the overall mean stands at 419 cases per 100,000 population. Age patterns exhibit peaks in both genders at ages 0–19 and 60 or older. An intense peak occurred every three years in 2010, 2013, and 2016. There was a noticeable variation among sub-districts, particularly in Songkhla and Pattani provinces.

**Fig 6:**
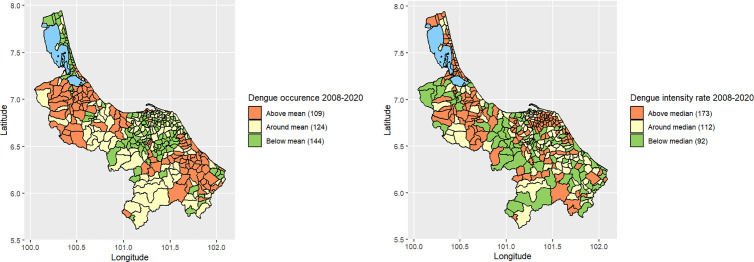
Maps of dengue occurrence (compared with mean percentage) and intensity (compared with median) by sub-districts in 2008-2020 in southernmost provinces of Thailand.

The 95% confidence interval of the predictors above, around, and below the overall mean was categorized into high, medium, and low occurrence or intensity groups based on the positioning of these intervals entirely above, around, or below a specified level. The sub-districts were classified based on their dengue occurrences and intensities, resulting in the creation of a thematic map. Fig 6 displays thematic maps illustrating the occurrence and intensity of dengue. The above mean occurrence indicates sub-districts that have a higher occurrence of dengue as compared to the mean percentage. The above median intensity of dengue indicates sub-districts that have a higher intensity of dengue as compared to the median. Most sub-districts in Songkhla and Narathiwat provinces experienced high occurrences, while the majority of sub-districts in Songkhla and Pattani had high intensity. Yala and Narathiwat also have some sub-districts with high spread.

**Fig 7:**
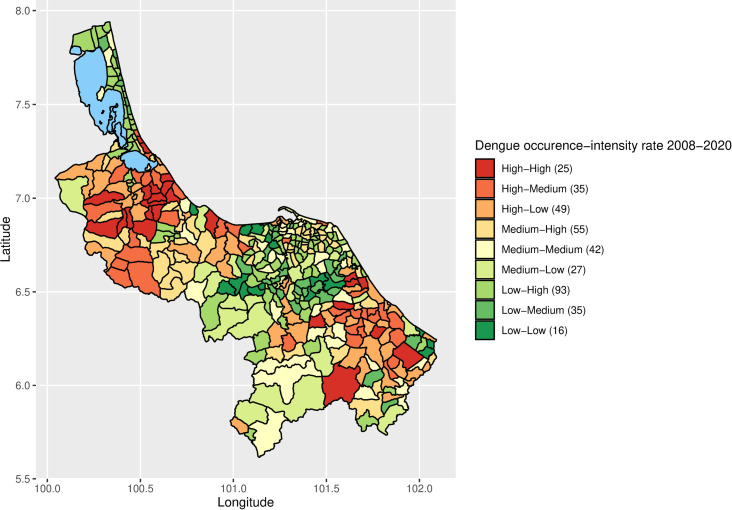
Occurrence-Intensity map of dengue in 2008-2020 in four southernmost provinces of Thailand.

Fig 7 displays a map illustrating the occurrence-intensity combinedly. This map displays a comprehensive range of occurrence-intensity levels. The occurrence levels and intensity rates were merged, leading to the formation of 9 categories: High-High, High-Medium, High-Low, Medium-High, Medium-Medium, Medium-Low, Low-High, Low-Medium, and Low-Low. The thematic map was then developed by classifying sub-districts according to the combined occurrence and intensity groups. The region on this map, where there was a significant occurrence and intensity of dengue, closely corresponds to the area depicted in Fig 1, where all sub-districts reported 150 or more cases over the span of 13 years. The dengue occurrence-intensity was high in most areas of Songkhla and Narathiwat whereas most areas in Pattani and Yala provinces had low dengue occurrence-intensity.

In Songkhla, seventeen sub-districts have experienced high occurrences and intensity of dengue. These sub-districts are located in Muang, Chana, Thepa, Hat Yai, Na Mom, Singhanakon, and Khlong Hoi Khong. Eight sub-districts in Narathiwat have a significant occurrence and intensity of dengue. These sub-districts are located in the districts of Bacho (Kayo Mati, Bare Nuea, and Bare Tai), Rangae (Tanyong Limo), Rueso (Samakkhi, Batong), Su-ngai Padi (Su-ngai Padi), and Chanae (Chang Phueak).

## Discussion

Occurrence-intensity model enables us to develop a method that can capture the unique incidence patterns associated with each outcome at different levels. Incidences are highest during young adulthood and decrease with age for both genders, while intensity is highest during young adulthood and old age. Three episodes of the dengue epidemic from 2008 to 2020 were found. The areas identified as high-risk mainly consisted of sub-districts in Songkhla and Narathiwat provinces.

Our study noticed that dengue was most prevalent among individuals aged 10–19. The result aligns with the findings of the Department of Disease Control, which reported that the age group 15–44 years old followed by infants aged 10–14 years was most susceptible to dengue fever infections from 2015 to 2019 [19]. In contrast, dengue incidence followed a V-shaped pattern, peaking after more than seventy years and collapsing after forty-nine years. This result is consistent with the findings of Van Benthem [20], who demonstrated that elderly individuals possessed limited dengue fever. Which rendered this age group more vulnerable to dengue fever than the others. The results revealed that dengue had three major epidemics occurred during the period of our study: in 2010, 2013, and 2016. This finding aligns with the recurrent dengue epidemics documented in Singapore [21] and Nepal [22]. The alteration in dengue can be plausibly attributed to the reduced duration of the rainy season or days with precipitation, coupled with the prolonged winter, particularly in 2017. This period was particularly unfavorable for mosquito breeding and disease transmission due to the La Niña and El Niño Southern Oscillation (ENSO) as suggested by Dostal et al. [23]. Dengue has decreased over the past two years, possibly because so many individuals remained at home during the COVID-19 pandemic when schools and offices were closed [24–25].

This analysis demonstrates that regions with a high risk of dengue can be identified using occurrence and intensity models. The study primarily identified regions with elevated dengue cases in Hat Yai, a city situated in the northern part of Songkhla province. Occasional outbreaks were also observed in areas along the province’s border with Narathiwat. The findings are similar to the findings of Rotejanaprasert et al. [26] and Xu et al. [27], which revealed that Songkhla is one of the top ten highest provinces and a hotspot in the southern area followed by the border area in Narathiwat. Our results also revealed that the occurrence-intensity was higher in sub-districts of Mueang Narathiwat. The findings are also endorsed by Boonklong and Bhumiratana [28] which suggested that Mueang Narathiwat is a higher urban-rural gradient area, so it is at more risk of having dengue cases. Virus transmission grows in tandem with human population density. In addition, urbanization in Hat Yai has resulted in the spread of *Aedes aegypti* as well as an increase in the number of vulnerable human hosts.

In this study, to evaluate the dengue’s occurrence and intensity, we considered only demographic variables to understand its pattern and suggest hotspots based on spatial distribution. We did not consider other environmental and health-related variables to explore their effect on dengue. Moreover, the availability of the data was till 2020, almost the start of COVID-19. We considered COVID-19 data in the analysis and did not make a difference between before and after COVID-19 as this pandemic is part of living. The proposed methods are subject to the limitations of a sufficiently large sample size, and the assumptions of each method must be satisfied. Additionally, the data used in these models was aggregated, so the methods are limited to individual cases. For further studies, researchers can use other environmental and health-related variables and may also consider more COVID-19 data to understand the distribution of dengue in these regions and use this methodology in other geographical areas. In addition, Other methods, such as machine learning methods, should be considered if the data does not conform to the assumptions.

## Conclusion

The proposed methodology based on the occurrence-intensity model can be seamlessly applied to other regions/countries, allowing for the spatial analysis of case counts over a significant duration. Moreover, this study highlighted the spatial patterns of occurrence and intensity of dengue disease in all sub-districts of the four most southern provinces of Thailand. By considering the high-risk areas of occurrence and intensity of dengue, healthcare experts can make decisions to mitigate the high occurrence and reduce the intensity in advance. Furthermore, with such strategies, public health experts can know where the high-risk areas are to monitor the spatial distribution and plan earlier control.

## Supporting information

S1 Dataset Dataset of dengue incidences. (XLSX)

## References

[1] World Health Organization. Dengue and severe dengue. 2023. Accessed 17 March 2023 https://www.who.int/news-room/fact-sheets/detail/dengue-and-severe-dengue

[2] Lim JK, Chanthavanich P, Limkittikul K, Lee J-S, Sirivichayakul C, Lee KS, et al. Clinical and epidemiologic characteristics associated with dengue fever in 2011-2016 in Bang Phae district, Ratchaburi province, Thailand. PLoS Negl Trop Dis. 2021;15(6):e0009513. doi: 10.1371/journal.pntd.0009513 34191799 PMC8244866

[3] Tejo AM, Hamasaki DT, Menezes LM, Ho YL. Severe dengue in the intensive care unit. J Intensive Med. 2023. 10.1016/j.jointm.2023.07.007 PMC10800775 38263966

[4] Johari NA, Voon K, Toh SY, Sulaiman LH, Yap IKS, Lim PKC. Sylvatic dengue virus type 4 in Aedes aegypti and Aedes albopictus mosquitoes in an urban setting in Peninsular Malaysia. PLoS Negl Trop Dis. 2019;13(11):e0007889. doi: 10.1371/journal.pntd.0007889 31730672 PMC6881067

[5] Messina JP, Brady OJ, Scott TW, Zou C, Pigott DM, Duda KA, et al. Global spread of dengue virus types: mapping the 70 year history. Trends Microbiol. 2014;22(3):138–46. doi: 10.1016/j.tim.2013.12.011 24468533 PMC3946041

[6] Cattarino L, Rodriguez-Barraquer I, Imai N, Cummings DAT, Ferguson NM. Mapping global variation in dengue transmission intensity. Sci Transl Med. 2020;12(528):eaax4144. doi: 10.1126/scitranslmed.aax4144 31996463

[7] Paz-Bailey G, Adams LE, Deen J, Anderson KB, Katzelnick LC. Dengue. The Lancet. 2024;403(10427):667–82. 10.1016/S0140-6736(23)02576-X PMC12372472 38280388

[8] Srichan P, Niyom SL, Pacheun O, Iamsirithawon S, Chatchen S, Jones C, et al. Addressing challenges faced by insecticide spraying for the control of dengue fever in Bangkok, Thailand: a qualitative approach. Int Health. 2018;10(5):349–55. doi: 10.1093/inthealth/ihy038 29912451 PMC6104709

[9] Rotejanaprasert C, Chinpong K, Lawson AB, Chienwichai P, Maude RJ. Evaluation and comparison of spatial cluster detection methods for improved decision making of disease surveillance: a case study of national dengue surveillance in Thailand. BMC Med Res Methodol. 2024;24(1):14. doi: 10.1186/s12874-023-02135-9 38243198 PMC10797994

[10] Lim JT, Dickens BS, Cook AR. Modelling the epidemic extremities of dengue transmissions in Thailand. Epidemics. 2020;33:100402. 32866907 10.1016/j.epidem.2020.100402

[11] Punyapornwithaya V, Sansamur C, Charoenpanyanet A. Epidemiological characteristics and determination of spatio-temporal clusters during the 2013 dengue outbreak in Chiang Mai, Thailand. Geospatial Health. 2020;15(2). 10.4081/gh.2020.857 33461277

[12] Rahman MS, Pientong C, Zafar S, Ekalaksananan T, Paul RE, Haque U, et al. Mapping the spatial distribution of the dengue vector Aedes aegypti and predicting its abundance in northeastern Thailand using machine-learning approach. One Health. 2021;13:100358. doi: 10.1016/j.onehlt.2021.100358 34934797 PMC8661047

[13] Abdulsalam FI, Antunez P, Yimthiang S, Jawjit W. Influence of climate variables on dengue fever occurrence in the southern region of Thailand. PLOS Global Public Health. 2022;2(4):e0000188. doi: 10.1371/journal.pgph.0000188 PMC10022128 36962156

[14] Rotejanaprasert C, Ekapirat N, Areechokchai D, Maude RJ. Bayesian spatiotemporal modeling with sliding windows to correct reporting delays for real-time dengue surveillance in Thailand. Int J Health Geogr. 2020;19(1):4. doi: 10.1186/s12942-020-00199-0 32126997 PMC7055098

[15] Polwiang S. The time series seasonal patterns of dengue fever and associated weather variables in Bangkok (2003-2017). BMC Infect Dis. 2020;20(1):208. doi: 10.1186/s12879-020-4902-6 32164548 PMC7068876

[16] Chumpu R, Khamsemanan N, Nattee C. The association between dengue incidences and provincial-level weather variables in Thailand from 2001 to 2014. PLoS One. 2019;14(12):e0226945. doi: 10.1371/journal.pone.0226945 PMC6932763 31877191

[17] Knerer G, Currie CSM, Brailsford SC. Reducing dengue fever cases at the lowest budget: a constrained optimization approach applied to Thailand. BMC Public Health. 2021;21(1):807. doi: 10.1186/s12889-021-10747-3 33906628 PMC8080389

[18] Tongkumchum P, McNeil D. Confidence intervals using contrasts for regression model. Songklanakarin J SciTech. 2009;31(2).

[19] Phanitchat T, Zhao B, Haque U, Pientong C, Ekalaksananan T, Aromseree S, et al. Spatial and temporal patterns of dengue incidence in northeastern Thailand 2006–2016. BMC Infectious Diseases. 2019;19:1–2. 31443630 10.1186/s12879-019-4379-3 PMC6708185

[20] Van Benthem BHB, Khantikul N, Panart K, Kessels PJ, Somboon P, Oskam L. Knowledge and use of prevention measures related to dengue in northern Thailand. Trop Med Int Health. 2002;7(11):993–1000. doi: 10.1046/j.1365-3156.2002.00950.x 12390606

[21] Rajarethinam J, Ang L-W, Ong J, Ycasas J, Hapuarachchi HC, Yap G, et al. Dengue in Singapore from 2004 to 2016: cyclical epidemic patterns dominated by serotypes 1 and 2. Am J Trop Med Hyg. 2018;99(1):204–10. doi: 10.4269/ajtmh.17-0819 29848407 PMC6085773

[22] Gyawali N, Johnson BJ, Dixit SM, Devine GJ. Patterns of dengue in Nepal from 2010–2019 in relation to elevation and climate. Transact Royal Soc Trop Med Hygiene. 2021;115(7):741–9. 10.1093/trstmh/traa131 33197254

[23] Dostal T, Meisner J, Munayco C, García PJ, Cárcamo C, Pérez Lu JE, et al. The effect of weather and climate on dengue outbreak risk in Peru, 2000-2018: a time-series analysis. PLoS Neglected Tropical Diseases. 2022;16(6):e0010479. doi: 10.1371/journal.pntd.0010479 PMC9278784 35771874

[24] Surendran SN, Nagulan R, Sivabalakrishnan K, Arthiyan S, Tharsan A, Jayadas TT, Raveendran S, Kumanan T, Ramasamy R. Reduced dengue incidence during the COVID-19 movement restrictions in Sri Lanka from March 2020 to April 2021. BMC Public Health. 2022 Feb 24;22(1):388. 35209890 10.1186/s12889-022-12726-8 PMC8866919

[25] Niriella MA, Ediriweera DS, De Silva AP, Premarathna BHR, Jayasinghe S, de Silva HJ. Dengue and leptospirosis infection during the coronavirus 2019 outbreak in Sri Lanka. Trans R Soc Trop Med Hyg. 2021;115(9):944–6. doi: 10.1093/trstmh/trab058 33823550 PMC8083582

[26] Rotejanaprasert C, Chinpong K, Lawson AB, Chienwichai P, Maude RJ. Evaluation and comparison of spatial cluster detection methods for improved decision making of disease surveillance: a case study of national dengue surveillance in Thailand. BMC Med Res Methodol. 2024;24(1):14. doi: 10.1186/s12874-023-02135-9 38243198 PMC10797994

[27] Xu Z, Bambrick H, Yakob L, Devine G, Lu J, Frentiu FD, et al. Spatiotemporal patterns and climatic drivers of severe dengue in Thailand. Sci Total Environ. 2019;656:889–901. doi: 10.1016/j.scitotenv.2018.11.395 30625675

[28] Boonklong O, Bhumiratana A. Seasonal and geographical variation of dengue vectors in Narathiwat, South Thailand. Canadian J Infect Diseases Med Microbiol. 2016;2016. 10.1155/2016/8062360 PMC4942596 27437001

